# Bacteriological profiling of diphenylureas as a novel class of antibiotics against methicillin-resistant *Staphylococcus aureus*

**DOI:** 10.1371/journal.pone.0182821

**Published:** 2017-08-10

**Authors:** Haroon Mohammad, Waleed Younis, Hany G. Ezzat, Christine E. Peters, Ahmed AbdelKhalek, Bruce Cooper, Kit Pogliano, Joe Pogliano, Abdelrahman S. Mayhoub, Mohamed N. Seleem

**Affiliations:** 1 Department of Comparative Pathobiology, Purdue University College of Veterinary Medicine, West Lafayette, Indiana, United States of America; 2 Department of Microbiology, Faculty of Veterinary Medicine, South Valley University, Qena, Egypt; 3 Department of Organic Chemistry, College of Pharmacy, Al-Azhar University, Cairo, Egypt; 4 Division of Biological Sciences, University of California, San Diego, La Jolla, California, United States of America; 5 Bindley Bioscience Center, Purdue University, West Lafayette, Indiana, United States of America; 6 Biomedical Sciences, University of Science and Technology, Zewail City of Science and Technology, Giza, Egypt; 7 Purdue Institute for Inflammation, Immunology, and Infectious Diseases, West Lafayette, Indiana, United States of America; Cornell University, UNITED STATES

## Abstract

Bacterial resistance to antibiotics remains an imposing global public health challenge. Of the most serious pathogens, methicillin-resistant *Staphylococcus aureus* (MRSA) is problematic given strains have emerged that exhibit resistance to several antibiotic classes including β-lactams and agents of last resort such as vancomycin. New antibacterial agents composed of unique chemical scaffolds are needed to counter this public health challenge. The present study examines two synthetic diphenylurea compounds **1** and **2** that inhibit growth of clinically-relevant isolates of MRSA at concentrations as low as 4 µg/mL and are non-toxic to human colorectal cells at concentrations up to 128 μg/mL. Both compounds exhibit rapid bactericidal activity, completely eliminating a high inoculum of MRSA within four hours. MRSA mutants exhibiting resistance to **1** and **2** could not be isolated, indicating a low likelihood of rapid resistance emerging to these compounds. Bacterial cytological profiling revealed the diphenylureas exert their antibacterial activity by targeting bacterial cell wall synthesis. Both compounds demonstrate the ability to resensitize vancomycin-resistant *Staphylococcus aureus* to the effect of vancomycin. The present study lays the foundation for further investigation and development of diphenylurea compounds as a new class of antibacterial agents.

## Introduction

Antibiotics have been critical therapeutic allies for healthcare-providers to treat bacterial infections for over 80 years. However, the increasing prevalence of clinical isolates of bacteria exhibiting resistance to one or more classes of antibiotics poses a significant global public health threat. A recent report found more than 60% of infectious disease physicians surveyed have treated at least one patient with a bacterial infection that was resistant to all commercially-available antibiotics [1]. In the United States of America alone, more than two million humans are afflicted with an antibiotic-resistant bacterial infection each year, resulting in 23,000 deaths [2]. Remarkably, a single bacterial pathogen, methicillin-resistant *Staphylococcus aureus* (MRSA), is responsible for nearly half of these fatalities. MRSA has been linked to both superficial skin infections [3, 4] and invasive diseases including osteomyelitis [5] and pneumonia [6]. A challenging aspect of treating these infections is clinical isolates of MRSA have emerged that exhibit resistance to multiple antibiotic classes including β-lactams [7], macrolides [8], quinolones [9, 10], tetracyclines [11], lincosamides [11], and mupirocin [11–13]. Further compounding this issue, strains of *S*. *aureus* have been isolated that exhibit resistance to antibiotics once deemed agents of last resort, including vancomycin (commonly referred to as vancomycin-resistant *S*. *aureus* or VRSA) [14, 15] and linezolid [16].

The emergence of bacterial resistance to current antibiotics necessitates the discovery and development of novel antibacterial agents. However, the field of antibiotic drug discovery has been severely hindered by the divestment of a number of large pharmaceutical companies. As of 2013, only four major pharmaceutical companies have active antimicrobial drug discovery programs [17, 18]. Not surprisingly, as the number of companies involved in antibacterial drug discovery has decreased, the number of new antibiotics introduced clinically has also plummeted from 29 newly approved antibiotics in the 1980s to just nine new antibiotics from 2000–2010 [19]. Remarkably, no new antibiotic class (defined as agents with distinct chemical structures or scaffolds) was introduced into the clinic from 1962 until 2000 [20]. Presently, all antibiotics in use today, including several of the most recently approved antibiotics, such as oritavancin (glycopeptide) and tedizolid phosphate (oxazolidinone), are derivatives of existing antibiotics discovered by 1984 [20]. Though several of these newer agents address key limitations of the parent drug, including enhancing the spectrum of activity against different bacterial species and reducing undesirable side effects, their similarity in structure to the parent drug often renders them susceptible to the same resistance mechanisms [20]. This highlights the need to identify antibacterial agents bearing new, previously unexploited chemical scaffolds.

**Fig 1:**
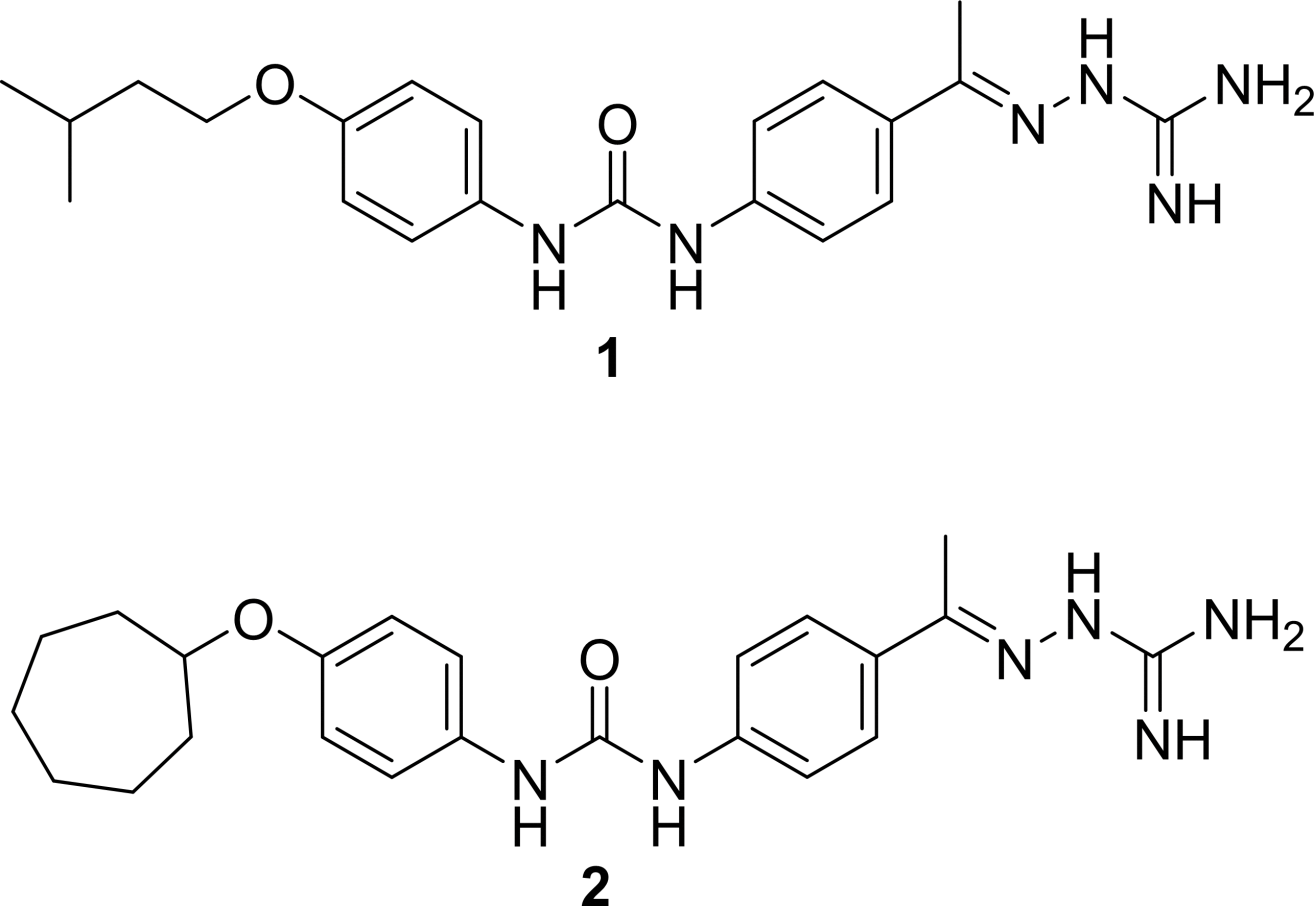
Chemical structures of diphenylurea compounds 1 and 2.

In order to identify novel antibacterial compounds bearing a unique scaffold, intensive *in silico* screening, following by pharmacokinetic profiling and several structural optimizations were conducted, as previously reported [21]. This subsequently led to the discovery of diphenylurea compounds **1** and **2** (Fig 1) that exhibited potent antibacterial activity against MRSA. The efficacy of these antibacterial compounds was validated in a *Caenhorhabditis elegans* model of MRSA infection where compound **2** proved superior to vancomycin in reducing the burden of MRSA in infected worms [21]. The present study builds upon this initial work by addressing several key unresolved questions including examining the antibacterial activity of **1** and **2** against a wider panel of drug-resistant *S*. *aureus* strains, the likelihood of MRSA to develop resistance to the diphenylurea compounds, the antibacterial mechanism of action of the diphenylurea compounds, and examining the compounds’ activity against staphylococcal biofilms. The results garnered from this study provide critical information to further develop this new class of antibacterial compounds.

## Results and discussion

### Diphenylurea compounds 1 and 2 are potent, bactericidal agents against MRSA and VRSA

The antibacterial activity of compounds **1** and **2** was examined against a panel of clinically-relevant strains of MRSA and VRSA, utilizing the broth microdilution assay. Of note, MRSA NRS384 (USA300) and MRSA NRS123 (USA400) are responsible for most MRSA infections in the United States [22] and specific regions in Canada [23], respectively. As presented in Table 1, the lead compound **1** inhibited growth of MRSA isolates consistently at a concentration of 4 μg/mL while compound **2** inhibited growth of the same isolates at concentrations ranging from 8 to 16 μg/mL. Vancomycin inhibited growth at concentrations ranging from 0.5 to 1 μg/mL. Interestingly, diphenylurea compounds **1** and **2** retained their antibacterial activity against strains of *S*. *aureus* exhibiting high-level resistance to the antibiotics mupirocin (NRS107) and vancomycin (VRS4, VRS7, VRS10 and VRS11a). Additionally, **1** and **2** exhibited potent activity against clinical isolates of MRSA that are resistant to multiple classes of antibiotics including ansamycins (NRS107), β-lactams, macrolides (NRS384 and NRS483), tetracyclines (NRS384), and fluoroquinolones (NRS387), indicating cross-resistance between these antibiotic classes and the diphenylurea compounds is unlikely to occur.

**Table 1:** The minimum inhibitory concentration (MIC in μg/mL) and the minimum bactericidal concentration (MBC in μg/mL) of diphenylurea compounds 1 and 2 and a control antibiotic (vancomycin) screened against *S*. *aureus* and *S*. *epidermidis* isolates.

	1		2		Vancomycin	
*S*. *aureus* strain	MIC	MBC	MIC	MBC	MIC	MBC
*S*. *aureus* NRS107	4	4	8	8	1	2
*S*. *aureus* ATCC 6538^a^	4	ND^c^	8	ND	0.5	ND
MRSA NRS123 (USA400)	4	4	8	8	1	1
MRSA NRS384 (USA300)	4	4	8	16	0.5	0.5
MRSA NRS387 (USA800)	4	4	8	16	0.5	1
MRSA NRS483 (USA1000)	4	4	16	16	1	1
MRSA NRS484 (USA1100)	8	8	16	16	1	2
VRS4^b^	8	8	16	16	64	>64
VRS7^b^	8	8	16	16	64	>64
VRS10^b^	4	4	4	8	>64	>64
VRS11a^b^	4	4	8	8	>64	>64
*S*. *epidermidis* NRS101^a^	2	ND	4	ND	1	ND

^a^Biofilm-forming strain

^b^Vancomycin-resistant *S*. *aureus*

^c^ND = not determined

**Fig 2:**
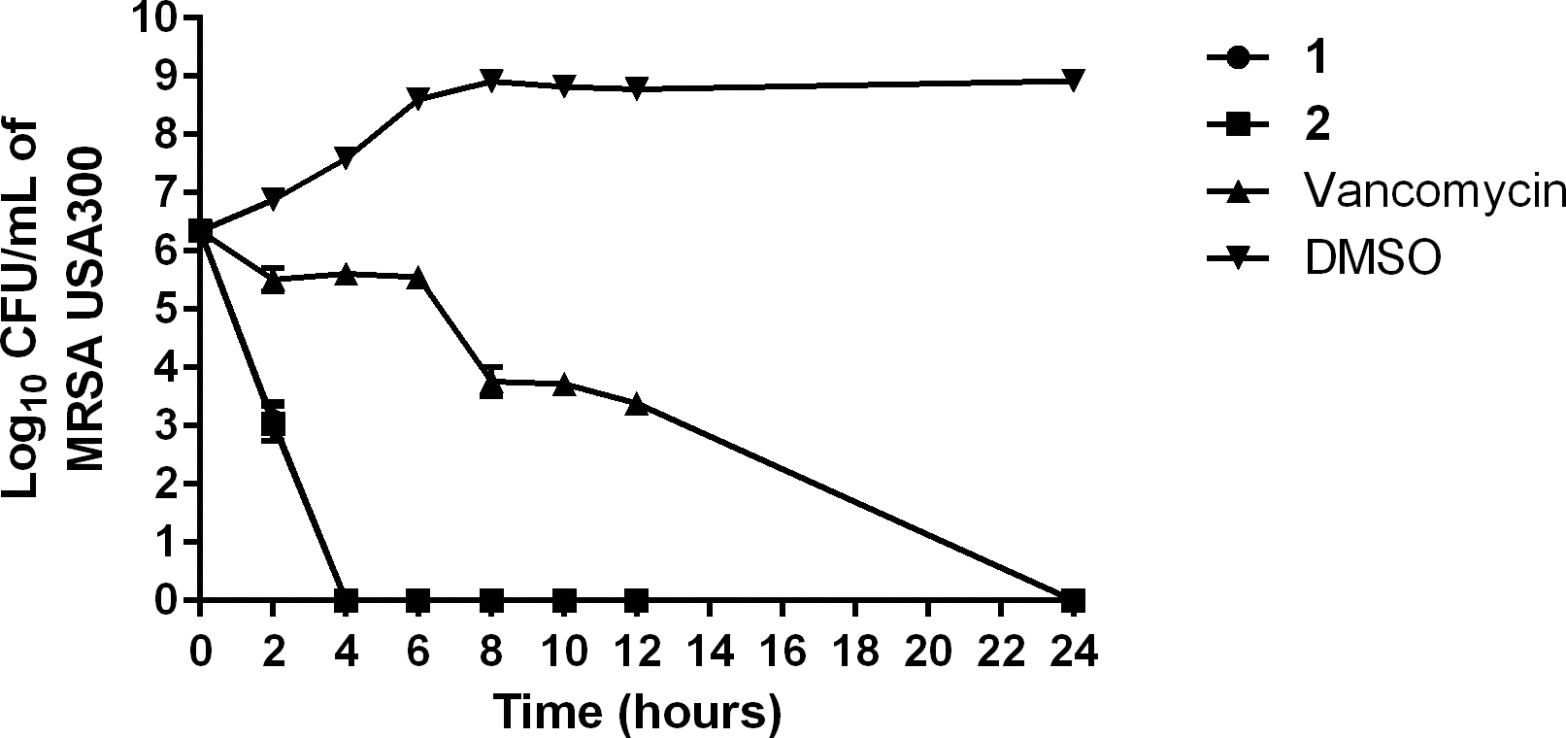
Time-kill assay of compounds 1, 2, and vancomycin (all at 4 × MIC) against methicillin-resistant *Staphylococcus aureus* (MRSA USA300). Test agents were incubated with MRSA over a 24 hour incubation period at 37 °C. DMSO served as a negative control. The error bars represent standard deviation values obtained from triplicate samples used for each compound/antibiotic studied.

We were curious to determine whether the diphenylurea compounds are bacteriostatic or bactericidal given bactericidal agents have been proposed to have certain advantages including helping patients to recover more rapidly from an infection and decreasing the emergence of resistance to the compound/antibiotic [24]. Thus, the minimum bactericidal concentration (MBC) was determined for both **1** and **2** against MRSA and VRSA. The MBC values for the diphenylurea compounds were found to be identical to or one-fold higher than the MIC values (Table 1). These results matched the results obtained with vancomycin, a known bactericidal antibiotic, suggesting the diphenylurea compounds are bactericidal agents. However to confirm this result more definitively, a time-kill assay was performed against MRSA USA300 (NRS384). As presented in Fig 2, both **1** and **2** (at 4 × MIC) reduce MRSA CFU/mL by 3-log_10_ within two hours, confirming the compounds are rapidly bactericidal against MRSA. Remarkably, both compounds completely eradicate a high inoculum of MRSA (~10^6^ CFU/mL) within four hours. Vancomycin exhibited slow bactericidal activity and required 24 hours to achieve the same effect, which is in agreement with previous reports [25, 26].

### Compounds 1 and 2 are non-toxic to mammalian cells at high concentrations

**Fig 3:**
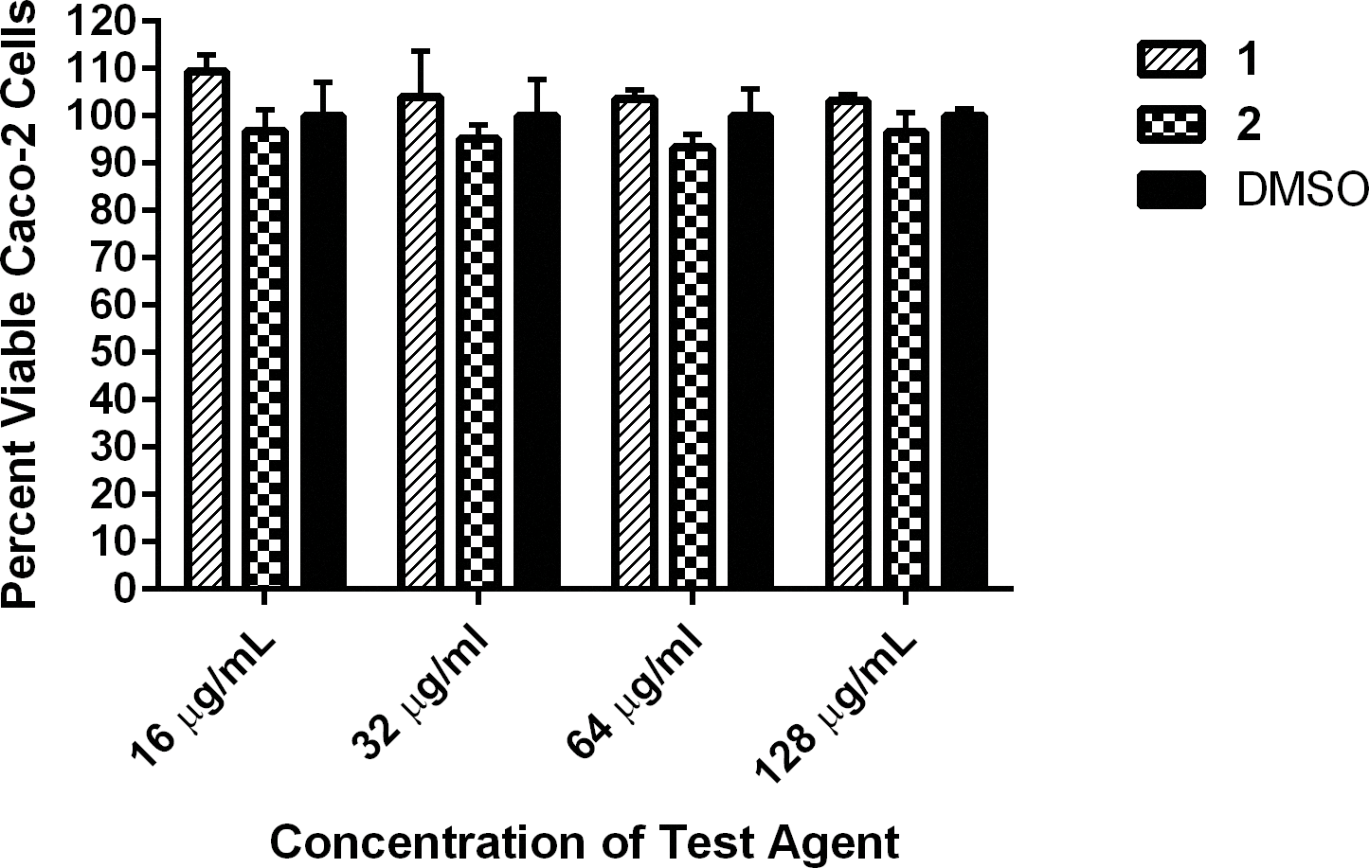
Toxicity analysis of diphenylurea compounds against human epithelial colorectal cells (Caco-2). Percent viable mammalian cells (measured as average absorbance ratio (test agent relative to DMSO)) for cytotoxicity analysis of diphenylurea compounds **1** and **2** (tested in triplicate) at 16, 32, 64, and 128 μg/mL against Caco-2 cells using the MTS assay. Dimethyl sulfoxide (DMSO) served as a negative control to determine a baseline measurement for the cytotoxic impact of each compound. The absorbance values represent an average of a minimum of three samples analyzed for each compound. Error bars represent standard deviation values for the absorbance values. A two-way ANOVA, with post hoc Dunnet’s multiple comparisons test, determined no statistical difference between the values obtained for each compound and DMSO (n = 3, *P* < 0.05).

Toxicity is a fundamental parameter to evaluate in early-stage drug discovery to ensure compounds with promising antibacterial activity do not also possess deleterious side effects to host (human) tissues. Previously, we evaluated the toxicity of compounds **1** and **2** against a human keratinocyte cell line. Compound **1** was found to be non-toxic to cells up to 32 μg/mL while **2** displayed an improved toxicity profile and was non-toxic to human keratinocytes up to a concentration of 64 μg/mL [21]. To further examine the toxicity profile of **1** and **2**, the MTS assay was utilized to evaluate both compounds against a human epithelial colorectal (Caco-2) cell line. We confirmed that both **1** and **2** were non-toxic up to 128 μg/mL, the highest concentration tested (Fig 3). This concentration is 31-fold higher than the MIC of **1** and 15-fold higher than the MIC of **2** against most strains of MRSA and VRSA examined. The results indicate both compounds have a promising safety profile that warrants further evaluation.

### MRSA mutants exhibiting resistance to compounds 1 and 2 could not be isolated

**Fig 4:**
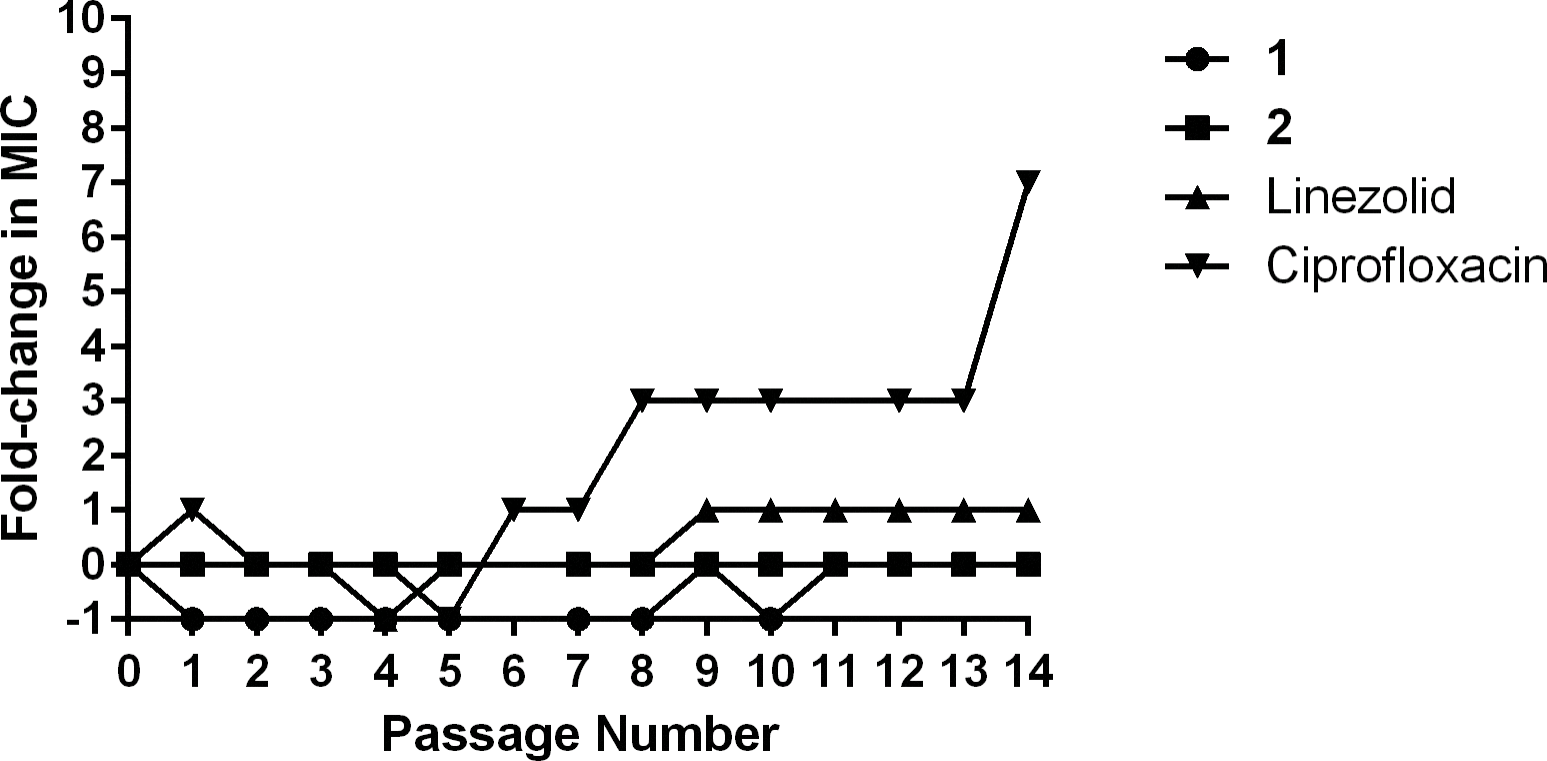
Multi-step resistance selection of compounds 1, 2, linezolid, and ciprofloxacin against methicillin-resistant *S*. *aureus* USA400 (NRS123). Bacteria were serially passaged over a 14-day period and the broth microdilution assay was used to determine the minimum inhibitory concentration of each compound against MRSA after each successive passage. A four-fold shift in MIC would be indicative of bacterial resistance to the test agent.

To assess the potential for rapid emergence of resistance of MRSA to the diphenylurea compounds, a multi-step resistance selection experiment was conducted. Initially the MICs of compounds **1** and **2** and control antibiotics with different resistance profiles (linezolid and ciprofloxacin) were determined against MRSA USA400 (NRS123) using the broth microdilution method and were found to be 8 μg/mL (compounds **1** and **2**), 2 μg/mL (linezolid), and 1 μg/mL (ciprofloxacin). Bacteria were then subcultured for fourteen passages over two weeks, to determine if a shift in the MIC of each agent tested would be observed. No increase in MIC was observed for either compound **1** or **2** after fourteen passages (Fig 4), indicating resistant mutants to these compounds could not be isolated. In contrast, a three-fold increase in the MIC for ciprofloxacin, a bactericidal antibiotic that interferes with bacterial DNA synthesis through inhibition of DNA gyrase [27], was found after just seven passages. The MIC for ciprofloxacin against MRSA continued to rise, increasing seven-fold after the fourteenth passage, indicating resistance had formed to this antibiotic. The rapid development of MRSA resistance to ciprofloxacin is in agreement with previous reports [28, 29]. The MIC of linezolid, a bacteriostatic antibiotic that inhibits bacterial protein synthesis, only increased one-fold over the fourteen passages, in agreement with a published study [30].

### Diphenylurea compounds target bacterial cell wall synthesis

**Fig 5:**
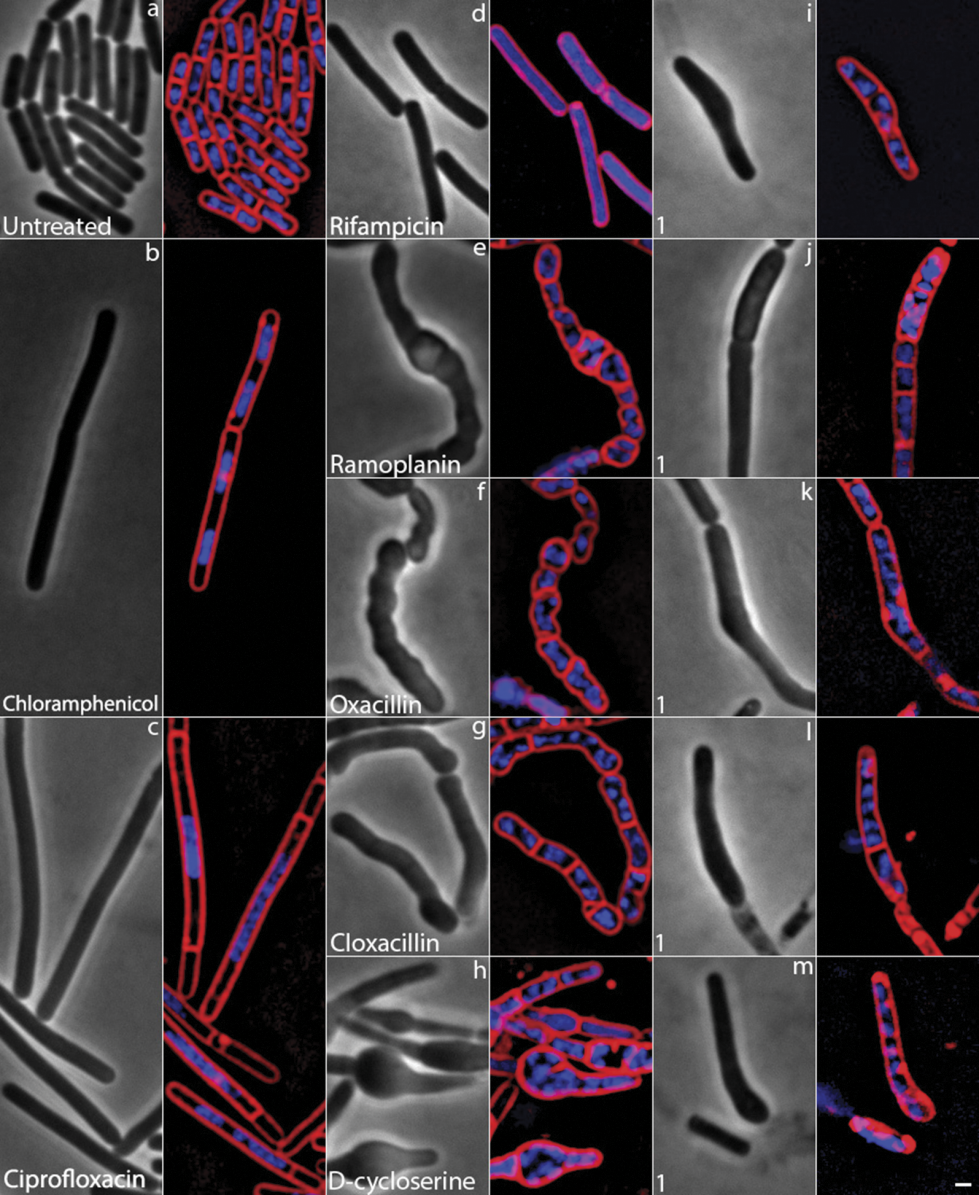
Bacterial cytological profiling of compound 1 against *Bacillus subtilis*. In *Bacillus subtilis* compound **1** induces cell shape defects similar to cell wall biosynthesis inhibitors. All cells were grown at 37 °C in LB-MSM and are shown at two hours. (**a**) Untreated cells. Treatment with (**b**) chloramphenicol at 5×MIC (10 μg/mL), (**c**) ciprofloxacin at 5×MIC (0.75 μg/mL), (**d**) rifampicin at 5×MIC (0.25 μg/mL), (**e**) ramoplanin at 1×MIC (0.375 μg/mL), (**f**) oxacillin at 5×MIC (1.87 μg/mL), (**g**) cloxacillin at 1×MIC (0.46 μg/mL), (**h**) D-cycloserine at 1×MIC (37.5 μg/mL), (**i-m**) Cells treated with compound **1** at 2.5×MIC (7.5 μg/mL). Compound **1** shows subtle cell shape defects consistent with cell wall inhibition. Cells are stained with FM 4−64 (red), DAPI (blue), and SYTOX Green (green). Scale bar is 1 μm.

The potent antibacterial activity of compounds **1** and **2** against MRSA and VRSA combined with the inability to isolate MRSA mutants exhibiting resistance to the diphenylureas led us to next investigate one of the most challenging questions in drug discovery–what is the antibacterial mechanism of action of these compounds? We investigated the mechanism of action of compound **1** using Bacterial Cytological Profiling (BCP) [31–33]. BCP identifies the likely pathway targeted by novel antibacterial agents by comparing their cytological effects with those found using a library of cytological profiles generated by using antibacterials with known mechanisms of action (MOAs), or by the rapid proteolytic depletion of essential proteins [31–33]. *Bacillus subtilis*, a representative Gram-positive bacterium, was utilized in this experiment, as BCP has not yet been developed for *S*. *aureus*. Using BCP, we identified that cells treated with compound **1** for two hours exhibited similar morphological features as cells treated with known cell wall active antibiotics. We compared **1** to known cell wall active antibiotics, such as cloxacillin, D-cycloserine, and ramoplanin in the presence of methylsulfonylmethane (MSM), which osmotically stabilizes cells for better observation of cell shape defects. MSM suppresses cell lysis and permeability defects for cell wall active antibiotics, but not for membrane active compounds [32]. Cells treated with **1** had cell shape defects after two hours in osmotically buffered media (Fig 5I, 5K and 5M), and formed small bulges, were misshapen, and appeared swollen (Fig 5L). These cells appeared similar to cells treated with cell wall active compounds such as ramoplanin (Fig 5E), oxacillin (5f) and D-cycloserine (Fig 5H), which also led to bulges and misshapen cells, and cloxacillin, which formed bulges at the poles of the cells (Fig 5G). Cells treated with compound **1** were dissimilar to cells treated with compounds targeting other pathways [31–33] including chloramphenicol, which inhibits protein synthesis, ciprofloxacin, which inhibits DNA replication, and rifampicin, a known transcription inhibitor (Fig 5B–5D). These results suggest that **1** inhibits cell wall biogenesis.

**Fig 6:**
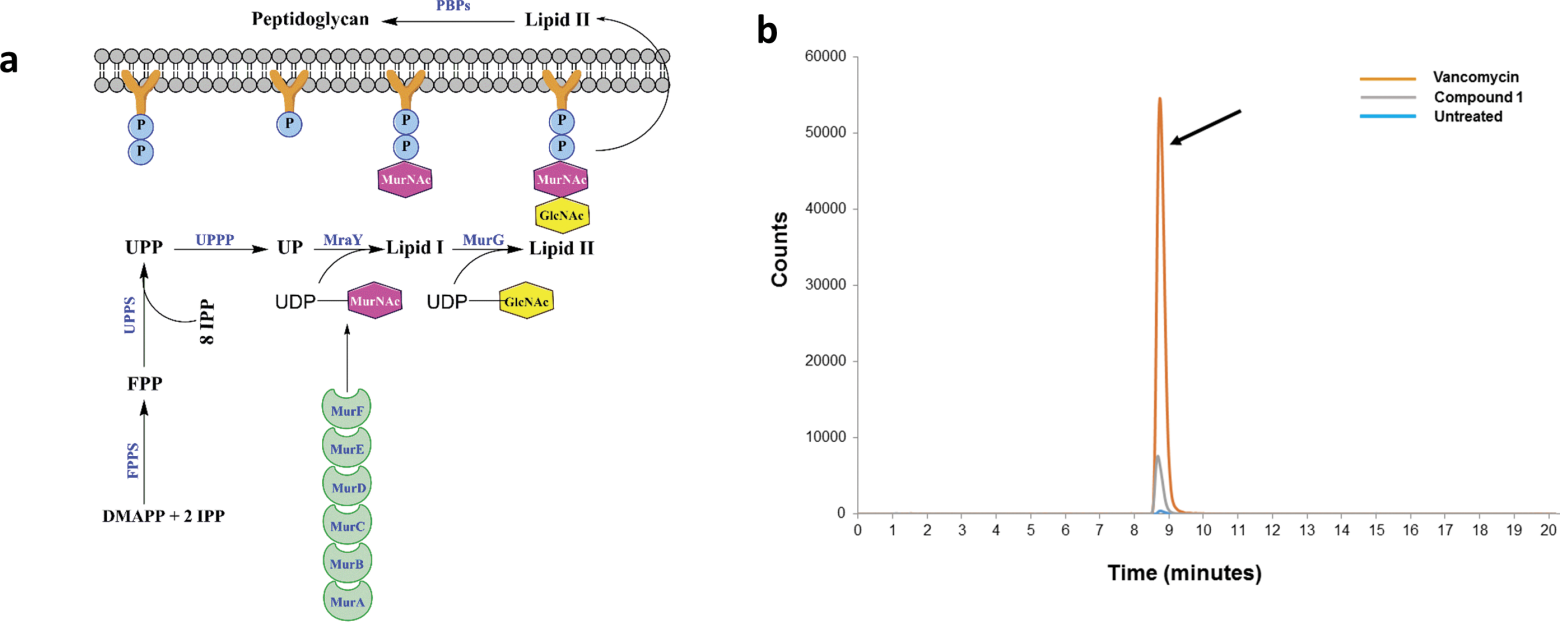
Simplified pathway for bacterial cell wall synthesis. **(a)** Diagram of key reactions in peptidoglycan synthesis for bacterial cell wall. Abbreviations: MurA, UDP-*N*-acetylglucosamine enolpyruvyl transferase; MurB, UDP-*N*-acetylenolpyruvylglucosamine reductase; MurC, UDP-*N*-acetylmuramoyl-L-alanine synthetase; MurD, UDP-*N*-acetylmuramyl-l-alanine:d-glutamate ligase; MurE, UDP-*N*-acetylmuramoyl-l-alanyl-d-glutamate:meso-2,6-diaminopimelate ligase, MurF, UDP-*N*-acetylmuramoyl-tripeptide-D-alanyl-D-alanine ligase, MurG, UDP-*N*-acetylglucosamine-*N*-acetylmuramyl-(pentapeptide) pyrophosphoryl-undecaprenol *N*-acetylglucosamine transferase; IPP, isopentenyl diphosphate, DMAPP, dimethylallyl diphosphate; FPP, isoprenoid farnesyl diphosphate; FPPS, farnesyl diphosphate synthase; UPP, C_55_ isoprenoid undecaprenyl diphosphate; UPPS, undecaprenyl diphosphate synthase; UPPP, undecaprenyl diphosphate phosphatase; UP, undecaprenyl monophosphate; MraY, phospho-MurNAc-pentapeptide translocase; GlcNAc, *N*-acetylglucosamine; MurNAc, *N*-acetylmuramic acid; PBPs, penicillin-binding proteins. **(b)** Detection of final soluble cell wall precursor (UDP-*N*-acetylmuramyl pentapeptide) inside bacterial cytoplasm. HPLC chromatogram of *S*. *aureus* NRS107 (RN4220) treated with 10 × MIC of compound **1** or vancomycin for 30 minutes. After centrifugation, the bacterial pellet was boiled for 30 minutes to release contents present in the bacterial cytoplasm. The lysate was analyzed using HPLC/MS, using a phenyl column, to determine the accumulation of the final soluble precursor in cell wall synthesis, UDP-*N*-acetylmuramyl pentapeptide (designated by the black arrow).

A major component of the bacterial cell wall in Gram-positive bacteria is a thick layer of peptidoglycan. Peptidoglycan is a unique structure only present in prokaryotic cells thus making this structure an excellent target for antibacterial drug discovery. Peptidoglycan is composed of linear glycan chains (alternating units of *N*-acetylglucosamine and *N*-acetylmuramic acid) interlinked by short peptides [34]. Synthesis of peptidoglycan is an intricate process that involves several key steps, as demonstrated in the simplified metabolic pathway in Fig 6A. First, UDP-*N*-acetylmuramyl pentapeptide is generated through a series of reactions that take place in the cytoplasm, catalyzed by the enzymes MurA, MurB, MurC, MurD, MurE, and MurF [34]. In a separate pathway, two molecules of isopentenyl diphosphate (IPP) combine with dimethylallyl diphosphate (DMAPP) to form the (C_15_) isoprenoid farnesyl diphosphate (FPP) [35]. This step is catalyzed by farnesyl diphosphate synthase (FPPS), with IPP/DMAPP produced by the mevalonate pathway in *S*. *aureus* and the non-mevalonate (methylerythritol phosphate, MEP) pathway in *B*. *subtilis* [35]. FPP then reacts with eight additional IPP molecules to form the (C_55_) isoprenoid undecaprenyl diphosphate (UPP) in a reaction catalyzed by undecaprenyl diphosphate synthase (UPPS) [36]. The enzyme undecaprenyl diphosphate phosphatase (UPPP) then cleaves a phosphate group from UPP to generate undecaprenyl monophosphate (UP) [37]. UP subsequently combines with UDP-*N*-acetylmuramyl pentapeptide, generated earlier in the cytoplasm, to form the essential lipid carrier Lipid I, in a reaction catalyzed by MraY [38]. A unit of *N*-acetylglucosamine (GlcNAc) is incorporated from uridine diphosphate *N*-acetylglucosamine to Lipid I to form Lipid II, in a reaction catalyzed by MurG [39]. Lipid II transports *N*-acetylmuramic acid (MurNAc) linked to *N*-acetylglucosamine across the cell membrane to the periplasmic space where it is eventually incorporated into the growing chain of peptidoglycan through a reaction catalyzed by the penicillin-binding proteins (PBPs, DD-transpeptidases) [39]. Antibiotics including ampicillin and vancomycin inhibit the later stages in cell wall synthesis (transpeptidation), by interfering with crosslinking of peptidoglycan chains, resulting in defects in cell wall structure [40].

UDP-*N*-acetylmuramyl pentapeptide is the final soluble precursor of cell wall synthesis that is generated in the bacterial cytoplasm. Therefore agents that target bacterial cell wall synthesis will lead to accumulation of this pentapeptide inside the bacterial cytoplasm which can be detected using HPLC-MS. Thus, in order to confirm the diphenylureas do exert their antibacterial effect by inhibiting cell wall synthesis in staphylococci, we measured the accumulation of UDP-*N*-acetylmuramyl pentapeptide in *S*. *aureus* NRS107 cells treated with either compound **1** or vancomycin. Cells treated with compound **1** and vancomycin resulted in a notable increase in UDP-*N*-acetylmuramyl pentapeptide accumulation (Fig 6B), implicating inhibition of peptidoglycan biosynthesis. A peak was present in the chromatogram at the same retention time (8.76 minutes) for both **1** and vancomycin-treated samples, and had the correct mass-to-charge ratio (*m/z*) for the pentapeptide, *m/z* = 1150.3588, a <1 ppm error. The intensity of the peak for vancomycin was notably higher than for **1**. We suspect this difference is due to two factors–the high concentration of test agent and the speed with which the test agents exert their antibacterial effect. As noted by the time-kill assay, compound **1** rapidly kills *S*. *aureus* in contrast to vancomycin. Thus we suspect the lower peak intensity for **1** is due to rupturing/lysing of some cells due to the high concentration (10 × MIC) used. This would decrease the amount of pentapeptide present in the cytoplasm. The net increase of UDP-*N*-acetylmuramyl pentapeptide in the cytoplasm after exposure to **1** supports inhibition of a target(s) in the peptidoglycan biosynthesis pathway though the exact molecular target remains to be determined.

### Compounds 1 and 2 are able to resensitize VRSA to vancomycin

The emergence of vancomycin-resistant *S*. *aureus* isolates has added an additional layer of complexity to healthcare providers’ attempts to treat multidrug-resistant staphylococcal infections. Given vancomycin’s importance clinically in treating drug-resistant *S*. *aureus* infections, a strategy that has recently been investigated is suppressing resistance to an antibiotic (such as vancomycin) using a secondary compound [41, 42]. The discovery that the diphenylureas inhibit peptidoglycan synthesis led us to investigate the compounds’ ability to resensitize VRSA to the effect of vancomycin. A previous study by our group has demonstrated that compounds targeting cell wall synthesis are capable of resensitizing vancomycin-resistant *S*. *aureus* to the effect of vancomycin [43]. Thus we exposed two strains of VRSA to subinhibitory concentrations (½ × MIC) of either compounds **1** or **2** for a short duration (30 minutes) before determining the MIC of vancomycin against both strains. As presented in Table 2, compound **1** (produces a 127-fold improvement in vancomycin MIC) is superior to **2** in re-sensitizing VRS4 to the effect of vancomycin while **2** proves superior to **1** in sensitizing VRS10 to the effect of vancomycin (63-fold improvement in MIC of vancomycin). Thus, the diphenylurea compounds do appear to have the ability to resensitize VRSA to the effect of vancomycin. The exact mechanism behind how the diphenylureas are able to resensitize VRSA to vancomycin is not yet resolved. However, antisense constructs targeting genes involved in early steps of cell wall synthesis in the bacterial cytoplasm, including the formation of UDP-*N*-acetylmuramyl pentapeptide (*murA*, *murB*, *murC*, *murD*, and *murE*), lipid I (*mraY*), and lipid II (*murG*), have been shown to increase the susceptibility of MRSA to the effect of antibiotics (including β-lactam antibiotics and vancomycin) that target latter stages of cell wall synthesis (transpeptidation) [44]. The accumulation of UDP-*N*-acetylmuramyl pentapeptide in *S*. *aureus* in the presence of **1**, as noted above, combined with the ability of **1** and **2** to resensitize VRSA to vancomycin suggests the diphenylurea compounds may target an essential enzyme involved in the early steps of peptidoglycan synthesis. However, additional investigation is needed to confirm this hypothesis.

**Table 2:** The minimum inhibitory concentration (MIC in μg/mL) of vancomycin in the absence and presence of a subinhibitory concentration (½ × MIC) of diphenylurea compounds 1 or 2 against vancomycin-resistant *S*. *aureus* (VRSA) isolates.

VRSA strain	MIC of vancomycin	MIC of vancomycin + 1	Fold re-sensitization of VRSA to vancomycin	MIC of vancomycin + 2	Fold re-sensitization of VRSA to vancomycin
VRS4	512	4	127-fold	64	7-fold
VRS10	512	128	3-fold	8	63-fold

### Diphenylurea compounds impact against staphylococcal biofilm

Patients that contract a staphylococcal infection are susceptible to recurring infections, even after repeated treatment with different antibiotics. The recurring infections are oftentimes due to the formation of staphylococcal biofilms near the site of initial infection or on the surface of medical devices inserted into patients (such as catheters or prosthetics) [45]. *Staphylococcus epidermidis* is the principal organism responsible for biofilm-associated infections, particularly in healthcare settings [46]. Bacterial cells present within biofilms typically exhibit increased resistance to conventional antibiotics because these agents are unable to effectively penetrate the biofilm [47]. Thus, agents capable of killing bacteria present within bacterial biofilm (to prevent recurrence of infection or dispersal of the biofilm to a new site) or disrupting adherent biofilm are highly desirable as they may have the ability to prevent the formation of chronic staphylococcal infections in afflicted patients.

**Fig 7:**
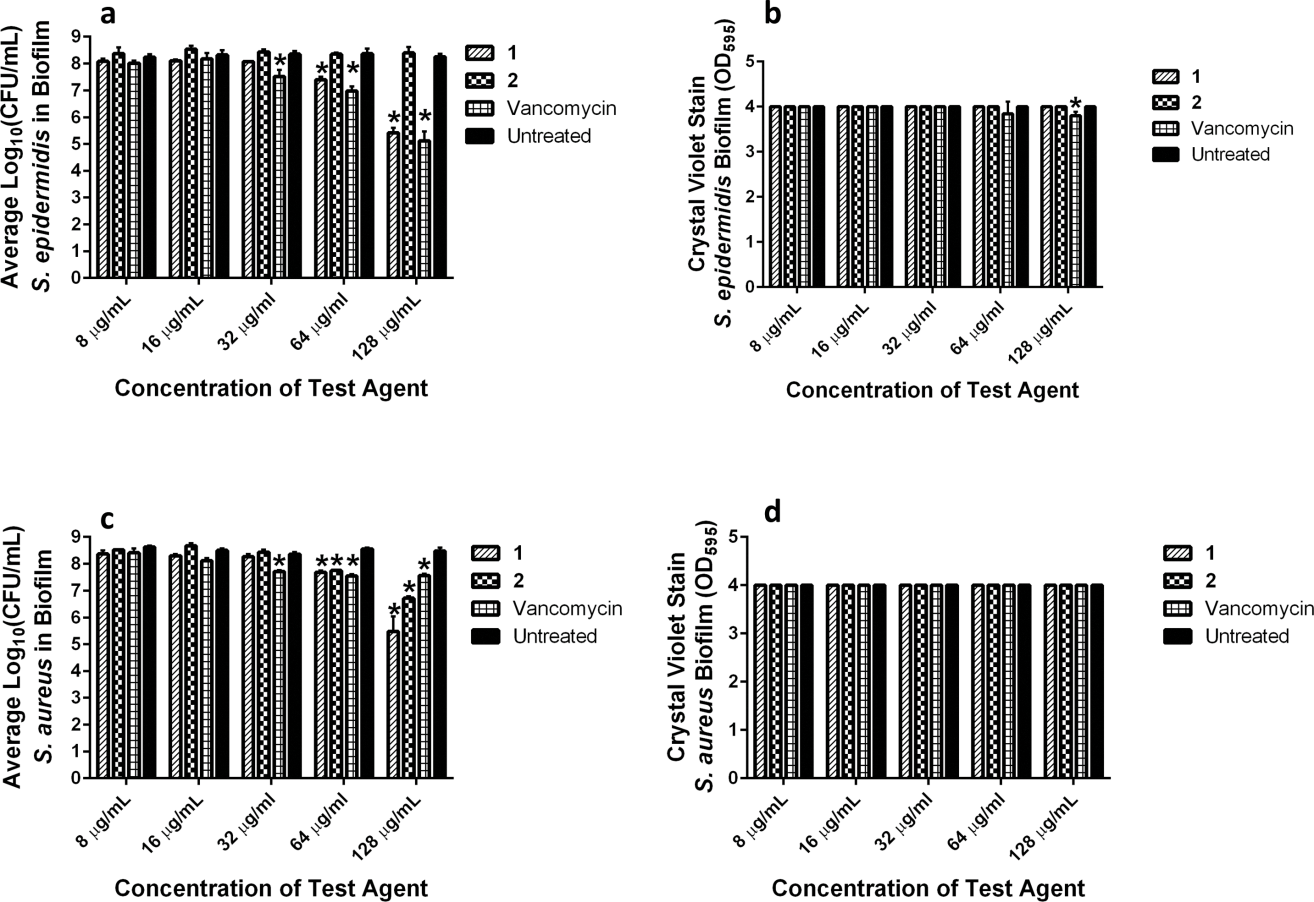
Examination of activity of diphenylurea compounds against mature staphylococcal biofilm. Detection of ability of diphenylurea compounds and vancomycin to **(a)** penetrate mature biofilm to inhibit/kill *S*. *epidermidis* NRS101 CFU present in biofilm and **(b)** disrupt integrity of mature biofilm of *S*. *epidermidis* NRS101 evaluated via crystal violet reporter assay. Detection of ability of diphenylurea compounds and vancomycin to **(c)** penetrate mature biofilm to kill *S*. *aureus* ATCC 6538 CFU present in biofilm and **(d)** disrupt mature biofilm of *S*. *aureus* ATCC 6538. Untreated wells served as a negative control. The values represent an average of a minimum of three samples analyzed for each compound/vancomycin. Error bars represent standard deviation values. Asterisk (*) denotes statistical significance between wells treated with compounds **1**, **2**, or vancomycin compared to untreated wells as determined by a two-way ANOVA with Dunnet’s multiple comparisons test (n = 3, *P* < 0.05).

Given *S*. *epidermidis* is the main culprit of staphylococcal biofilm infections clinically, we initially examined the ability of the diphenylurea compounds to penetrate *S*. *epidermidis* biofilm and reduce the burden of bacteria present inside. After treatment (24 hours) of *S*. *epidermidis* biofilm with compounds **1**, **2**, or vancomycin, the viable bacteria that survived treatment was determined. As presented in Fig 7A, compound **1** and vancomycin exerted a concentration-dependent reduction in bacterial burden within *S*. *epidermidis* biofilm. At concentrations of 8, 16, and 32 μg/mL, no reduction in bacterial CFU was observed for compound **1** when compared to the untreated control wells. However, at 64 μg/mL, both compound **1** (1.0-log_10_ reduction) and vancomycin (1.4-log_10_ reduction) significantly reduced bacterial CFU present within *S*. *epidermidis* biofilm. Remarkably, at 128 μg/mL, compound **1** generated a 2.84-log_10_ reduction in *S*. *epidermidis* CFU while vancomycin generated a 3.14-log_10_ reduction. Interestingly, compound **2** was unable to reduce *S*. *epidermidis* CFU at all concentrations tested (from 8 to 128 μg/mL). In order to examine whether the reduction in bacterial CFU caused by **1** and vancomycin was due to physical disruption of the integrity of the biofilm, mature *S*. *epidermidis* biofilm was treated with compounds **1**, **2**, or vancomycin. The biofilm mass, after 24-hour treatment with compounds **1**, **2**, or vancomycin, was quantified using the crystal violet reporter assay. As presented in Fig 7B, there was no decrease in the biofilm mass in the presence of compounds **1** or **2**, even at a concentration of 128 μg/mL. Similarly, at concentrations of 8 to 64 μg/mL, no significant reduction in biofilm mass was observed for vancomycin compared to the untreated control. A slight decrease in biofilm mass was observed for vancomycin (OD_595_ = 3.80) at 128 μg/mL relative to the untreated control (OD_595_ = 4.00). Overall, the results indicate compound **1** and vancomycin are capable of penetrating *S*. *epidermidis* biofilm and kill bacterial cells present within the biofilm, albeit at high concentrations. This reduction in bacterial CFU for compound **1** does not appear to be due to physical disruption of the biofilm integrity.

The results obtained against *S*. *epidermidis* biofilm led us to examine the ability of the diphenylurea compounds and vancomycin to kill bacteria present within *S*. *aureus* biofilm. A similar, concentration-dependent killing of *S*. *aureus* CFU present within the biofilm was observed for compound **1** and vancomycin, as observed against *S*. *epidermidis* (Fig 7C). At a concentration of 64 μg/mL, both compound **1** (0.9-log_10_ reduction) and vancomycin (1.0-log_10_ reduction) significantly reduced bacterial CFU present within *S*. *aureus* biofilm. The burden of *S*. *aureus* within the biofilm was further reduced when exposed to a higher concentration (128 μg/mL) of compound **1** (3.0-log_10_ reduction) but not vancomycin. Interestingly, compound **2**, which was ineffective at reducing *S*. *epidermidis* CFU in the biofilm, was able to reduce *S*. *aureus* CFU at concentrations of 64 μg/mL (0.8-log_10_ reduction) and 128 μg/mL (1.76—log_10_ reduction). We confirmed that the decrease in *S*. *aureus* CFU within the biofilm after exposure to compounds **1**, **2**, or vancomycin was not due to physical disruption of the biofilm mass via the crystal violet reporter assay. There was no change in the optical density values obtained for *S*. *aureus* biofilm treated with compounds **1**, **2**, or vancomycin at all concentrations tested (Fig 7D).

### Assessment of physicochemical properties of compounds 1 and 2

Thus far, results garnered for diphenylureas **1** and **2** indicated they are promising antibacterial agents. However, to effectively examine these compounds in suitable animal models of MRSA infection, the physicochemical properties must first be characterized. Properties such as aqueous solubility, permeability, stability to hepatic metabolism, and potential binding of compound/drug to proteins present in serum play a key role in determining suitable route(s) of administration (and what types of infection can possibly be treated such as local skin lesions versus systemic infections) [48].

We assessed the physicochemical properties of **1** and **2** by examining their aqueous solubility, permeability (ability to cross the gastrointestinal tract), stability to hepatic metabolism, and binding to human serum albumin (HSA). Previously, we found the compounds exhibited poor permeability using the well-established Caco-2 bidirectional permeability assay [21]; this suggested oral administration of the diphenylureas would not be suitable as the compounds would not be expected to cross the GI tract and accumulate at a clinically achievable concentration in blood. Both **1** and **2** exhibited highly acceptable initial metabolic stability profiles when tested with human liver microsomes (intrinsic half-life exceeded four hours) [21], indicating injectable administration may be possible for treatment of systemic MRSA infections. To confirm intravenous administration would be a feasible route of delivery for the diphenylureas, we examined their aqueous solubility and ability to bind to human serum albumin, a major protein present in blood that reduces the free fraction of drug/compound available in circulation [49]. The MIC of compounds **1** and **2**, linezolid, and daptomycin against MRSA USA400 (NRS123) was determined in the presence and absence of a physiological concentration (4%) of human serum albumin (HSA). As presented in Table 3, both **1** and **2** do appear to bind to human serum albumin. A seven-fold increase in the MIC was observed for compound **1** and a 15-fold increase in MIC was observed for compound **2** against MRSA in the presence of HSA. The antibiotic daptomycin, a drug known to bind strongly to human serum albumin [50], exhibited a 31-fold increase in MIC when examined under the same experimental conditions. The MIC for linezolid, in contrast, remained unchanged in agreement with a previous report [51].

**Table 3:** The minimum inhibitory concentration (MIC in μg/mL) of diphenylurea compounds 1, 2 and control drugs in the presence of human serum albumin and examination of the compounds’ aqueous solubility limit.

Compound/Drug	MIC vs. MRSA USA400 (-HSA)^a^	MIC vs. MRSA USA400 (+HSA)^a^	Aqueous solubility limit^b^ (μg/mL)
**1**	4	32	23.95
**2**	8	128	5.15
Daptomycin	2	64	-
Linezolid	1	1	-
Tamoxifen	-	-	5.80
Verapamil	-	-	>227.30

^a^HSA = human serum albumin

^b^Solubility limit corresponds to the highest concentration of test compound where no precipitate was detected (OD_540_)

We also examined the highest concentration where compounds **1** and **2** would remain soluble in an aqueous solution (saline). Compound **1** remained soluble in an aqueous solution up to 23.95 μg/mL while compound **2** exhibited much poorer solubility (5.15 μg/mL) similar to the poorly soluble drug tamoxifen (5.80 μg/mL) (Table 3). The modest increase in MIC for compounds **1** and **2** in the presence of HSA and their poor aqueous solubility suggest that intravenous administration of the diphenylureas, in their present state, may not be suitable in part because a higher dose/concentration (possibly toxic to mammalian cells/tissues) would need to be administered to effectively kill MRSA. The limited physicochemical properties of the diphenylureas is not altogether surprising. Indeed, nearly 90% of drugs currently in the discovery/development pipeline exhibit limited physicochemical properties (namely poor solubility, poor permeability, or both) [52]. Thus, future directions involving the diphenylurea compounds will focus on designing analogues with improved drug-like properties (enhance solubility and/or permeability, decrease binding to human serum albumin, increase the safety margin/profile to host tissues) in order to examine their effectiveness in appropriate animal models of MRSA infection. This approach has been successfully employed by our research group to improve the therapeutic potential of other small molecule compounds with potent anti-MRSA activity [53], and we believe can also be achieved with the diphenylurea compounds.

## Conclusions

The present study confirms diphenylurea compounds **1** and **2** are potent inhibitors of MRSA and VRSA growth. MRSA mutants exhibiting resistance to either compound **1** or **2** could not be isolated even after repeated subculturing over fourteen passages, indicating a low likelihood of rapid resistance emerging. Closer investigation of the mechanism of action of the diphenylureas revealed they exert their antibacterial effect by interfering with bacterial cell wall synthesis. Interestingly, both compounds **1** and **2** are capable of re-sensitizing VRSA to the effect of vancomycin. Furthermore, compound **1** is able to penetrate both *S*. *aureus* and *S*. *epidermidis* mature biofilm to reduce the burden of bacteria present within the biofilm, albeit at high concentrations. The physicochemical properties of the diphenylurea compounds currently precludes investigating their efficacy in treating systemic MRSA infections in suitable animal models. Future studies will aim to address the current limitations of the diphenylurea compounds in order to facilitate their development as novel antibacterial agents for treatment of drug-resistant staphylococcal infections.

## Materials and methods

### Synthesis of diphenylurea compounds

Synthetic schemes, spectral data, and purity (>95%, determined by Elemental Analysis) of diphenylurea compounds **1** and **2**, in addition to all intermediates, have been reported elsewhere [21]. Both compounds were dissolved in dimethyl sulfoxide (DMSO) to prepare a stock (10 mg/mL) solution.

### Bacterial strains and reagents used in this study

Clinical isolates of *S*. *aureus* were obtained through the Network of Antimicrobial Resistance in *Staphylococcus aureus* (NARSA) program. Antibiotics were purchased commercially and dissolved in DMSO (for linezolid and rifampicin), ethanol (for chloramphenicol), 0.1N hydrochloric acid (for ciprofloxacin), or sterile deionized water (for daptomycin, D-cycloserine, cloxacillin, oxacillin, ramoplanin, and vancomycin). Stock 10 mg/mL solutions were prepared for all drugs except for cloxacillin (25 mg/mL), ciprofloxacin (25 mg/mL), and chloramphenicol (50 mg/mL). Cation-adjusted Mueller-Hinton broth (CAMHB), crystal violet, penicillin-streptomycin, Trypsin-EDTA, Tryptic soy broth (TSB), Tryptic soy agar (TSA), phosphate-buffered saline (PBS), Dulbeco’s modified Eagle’s medium (DMEM), fetal bovine serum (FBS), and 96-well plates were all purchased from commercial vendors.

### Determination of minimum inhibitory concentration (MIC) and minimum bactericidal concentration (MBC) against drug-resistant *S*. *aureus* strains

The broth microdilution assay was employed to determine the MIC of compounds **1**, **2**, and vancomycin against five MRSA strains, four VRSA strains, one highly mupirocin-resistant *S*. *aureus* strain, one biofilm-forming *S*. *aureus* (ATCC 6538) strain, and one biofilm-forming methicillin-resistant *S*. *epidermidis* strain, as per the guidelines of the Clinical and Laboratory Standards Institute (CLSI) [54]. Plates containing the bacterial suspension (in CAMHB) and test agents (at concentrations ranging from 64 μg/mL down to 0.5 μg/mL) were incubated at 37 °C for 19 hours before the MIC was determined by visual inspection. The MBC was determined by plating an aliquot (5 μL) from wells with no growth onto TSA plates. Plates were incubated at 37 °C for 19 hours before recording the MBC (concentration where no growth was observed on TSA plates).

### Time-kill assay of diphenylurea compounds against MRSA

MRSA USA400, in logarithmic growth phase (OD_600_ = 0.80), was diluted to 2.26 × 10^6^ colony-forming units (CFU/mL) and exposed to concentrations equivalent to 4 × MIC (in triplicate) of compounds **1**, **2**, and vancomycin, in TSB. Aliquots (100 μL) were collected from each treatment group after 0, 2, 4, 6, 8, 10, 12, and 24 hours of incubation at 37 °C and subsequently serially diluted in PBS. Bacteria were then transferred to TSA plates and incubated at 37 °C for 18–20 hours before viable CFU/mL was recorded.

### Cytotoxicity analysis of compounds 1 and 2 in cell culture

Compounds **1** and **2** were assayed (at concentrations of 16 μg/mL, 32 μg/mL, 64 µg/mL, and 128 μg/mL) against a human epithelial colorectal (Caco-2) cell line (American Type Culture Collection, ATCC HTB-37) using the MTS assay [26]. Cells were cultured in DMEM supplemented with penicillin-streptomycin, nonessential amino acids (1%), and FBS (10%), at 37 °C with CO_2_ (5%). The cells were incubated with compounds (in triplicate) or DMSO (negative control) in a 96-well tissue culture-treated plate at 37 °C with CO_2_ (5%) for two hours. The MTS assay reagent (Promega, Madison, WI, USA) was subsequently added to each well and plates were incubated for four hours at 37 °C with CO_2_ (5%). The quantity of viable cells (at OD_490_) after treatment was expressed as a percentage of the viability of DMSO-treated control cells (average of triplicate wells ± standard deviation). The toxicity data was analyzed via a two-way ANOVA, with post hoc Dunnet’s multiple comparisons test (n = 3, *P* < 0.05), utilizing GraphPad Prism 6.0 (GraphPad Software, La Jolla, CA).

### Multi-step resistance selection against MRSA

To assess MRSA’s ability to develop resistance to the diphenylurea compounds after repeated exposure, a multi-step resistance selection experiment was conducted [55]. The broth microdilution assay was utilized to determine the MIC of compounds **1** and **2**, linezolid, and ciprofloxacin exposed to MRSA USA400 (NRS123) for 14 passages over a period of two weeks. Resistance was classified as a greater than four-fold increase in the initial MIC, as reported elsewhere [56].

### Bacterial cytological profiling to determine antibacterial mechanism of action

*B*. *subtilis* cells were grown in Luria Bertani (LB) medium at 37 °C until the optical density at 600 nm (OD_600_) was ~0.20. Cells were then left untreated or treated with compound **1** or control antibiotics in the presence of methylsulfonylmethane (MSM), as described previously [31–33]. After two hours, cells were stained with FM 4−64 (1 μg/mL) to visualize cell membranes; DAPI (1 μg/ml) to visualize DNA, and SYTOX Green (1 μg/mL), a vital stain which is normally excluded from cells with an intact membrane but brightly stains cells that are lysed [33]. Images were collected using a Delta Vision Spectris Deconvolution microscope, as described previously [33].

### Detection of accumulation of UDP-N-acetylmuramyl pentapeptide

The accumulation of UDP-*N*-acetylmuramyl pentapeptide was detected using a procedure described in a previous study [57], with the following modifications. *S*. *aureus* NRS107 (RN4220), in early logarithmic growth stage (OD_600_ ~ 0.60), was incubated with 130 μg/mL chloramphenicol for 15 minutes at 37°C. Bacteria were subsequently incubated with either 10 × MIC of compound **1** (most potent diphenylurea compound against this strain) or vancomycin (positive control) for 30 minutes, at 37°C. Untreated samples served as a negative control. Samples were centrifuged at 10,000 rpm for five minutes, the supernatant discarded, and the pellet re-suspended in sterile deionzined water (1 mL). The pellet was boiled at 10°C for 30 minutes before samples were chilled on ice for 10 minutes. UDP-*N*-acetylmuramyl-pentapeptide was measured using an Agilent High Performance Liquid Chromatography coupled to a time-of-flight Mass Spectrometer (HPLC-MS). A Waters XBridge Phenyl (2.1 × 100 mm, 3.5 μm) chromatography column was used, with mobile phases of water, 0.1% formic acid (Buffer A) and acetonitrile, 0.1% formic acid (Buffer B). A gradient of 5–20% Buffer B over 14 minutes was used, with a flow rate of 0.3 mL/min. An electrospray source was used, in positive ionization mode. Extracted Ion Chromatograms (EIC) were generated at a mass-to-charge ratio (*m/z*) of 1150.3588 (20 ppm window). Mass error for UDP-*N*-acetylmuramyl-pentapeptide was <1 ppm.

### Resensitization of VRSA to vancomycin in the presence of diphenylurea compounds

CAMHB was inoculated with strain VRS4 or strain VRS10 (5×10^5^ CFU/mL), as described previously [58], with the following modifications. Aliquots (5 mL) of the bacterial suspension were divided into micro-centrifuge tubes and compounds **1** or **2** (at ½ × MIC) were introduced into each tube. After 30 minutes of incubation at room temperature, samples from each tube were transferred to a new micro-centrifuge tube, prior to addition of vancomycin (at 128 μg/mL). Plates containing the test agents and bacteria were then incubated at 37 °C for 20–22 hours after which the MIC value was determined. A fold-reduction was calculated by comparing the MIC of vancomycin compared to the MIC of vancomycin given in combination with compounds **1** or **2** (at ½ × MIC).

### Examination of diphenylurea compounds’ ability to disrupt mature staphylococcal biofilm

Compounds **1**, **2**, and vancomycin were examined for their ability to eradicate mature staphylococcal biofilm using the microtiter dish biofilm formation assay [59]. *S*. *epidermidis* NRS101 and *S*. *aureus* ATCC 6538 were selected as the test organisms given their ability to form strong adherent biofilm. An overnight culture of bacteria was diluted (1:100 in TSB + 1% glucose) and transferred to a 96-well plate. The plate was incubated for 24 hours at 37 °C in order to form adherent biofilm on the surface of the plate wells. The medium containing the bacterial suspension was removed and the biofilm was washed with sterile PBS to remove planktonic cells. Compounds **1**, **2**, or vancomycin (from 128 to 1 μg/mL), in triplicate, in TSB were incubated with the biofilm for 24 hours at 37 °C.

### Quantification of bacterial CFU inside biofilm post-treatment

For enumeration of viable CFU inside the biofilm, compounds were removed, wells were washed with sterile PBS, and treated with 0.25% Trypsin-2.21 mM EDTA solution as described elsewhere [60]. The solution containing cells was transferred to a 96-well plate, serially diluted in PBS, and plated on TSA. Plates were incubated at 37 °C for 19 hours before viable CFU were counted. Data are presented as average log_10_(CFU/mL) of bacteria present in the biofilm post-treatment.

### Quantification of biofilm mass disruption

Compounds were subsequently removed. The biofilm was washed with PBS and then stained with 1% (w/v) crystal violet for 40 minutes. The stain was removed and wells were washed with sterile PBS. Wells were next decolorized using 95% ethanol. The optical density of each well at 595 nm was measured using a microplate reader.

A two-way ANOVA with Dunnet’s multiple comparisons, (n = 3, *P* < 0.05), was used to determine statistical significance between wells treated with compounds **1**, **2**, or vancomycin compared to untreated wells.

### Examination of diphenylurea compounds binding affinity to human serum albumin

The broth microdilution assay presented above was used to assess if compounds **1** and **2** bind to a major component of blood (human serum albumin) thus limiting the free fraction of drug available to exert its antibacterial effect. Compounds and control antibiotics (testing concentration at a range of 128 μg/mL down to 1 μg/mL) were incubated with MRSA NRS123 (USA400), using TSB in the presence and absence of a physiological concentration (4%) of human serum albumin, in a 96-well plate at 37 °C for 23 hours before recording the MIC. CaCl_2_ (20 μg/mL) was added to wells containing daptomycin, per the CLSI guidelines.

### Aqueous solubility assessment for diphenylurea compounds

**S**erial dilutions of the tested compounds, tamoxifen, and verapamil were prepared in PBS at 100 × the final concentration. The solutions were diluted 100-fold into PBS in a 96-well plate and mixed. The absorbance of the PBS-containing plate was measured prior to addition of the test agents to determine the background absorbance. After two hours, the presence of precipitate was detected by turbidity (absorbance at 540 nm). An absorbance value of greater than mean + 3 × standard deviation of the blank, after subtracting the pre-experiment background, is indicative of turbidity. The solubility limit is reported as the highest experimental concentration with no evidence of turbidity.
